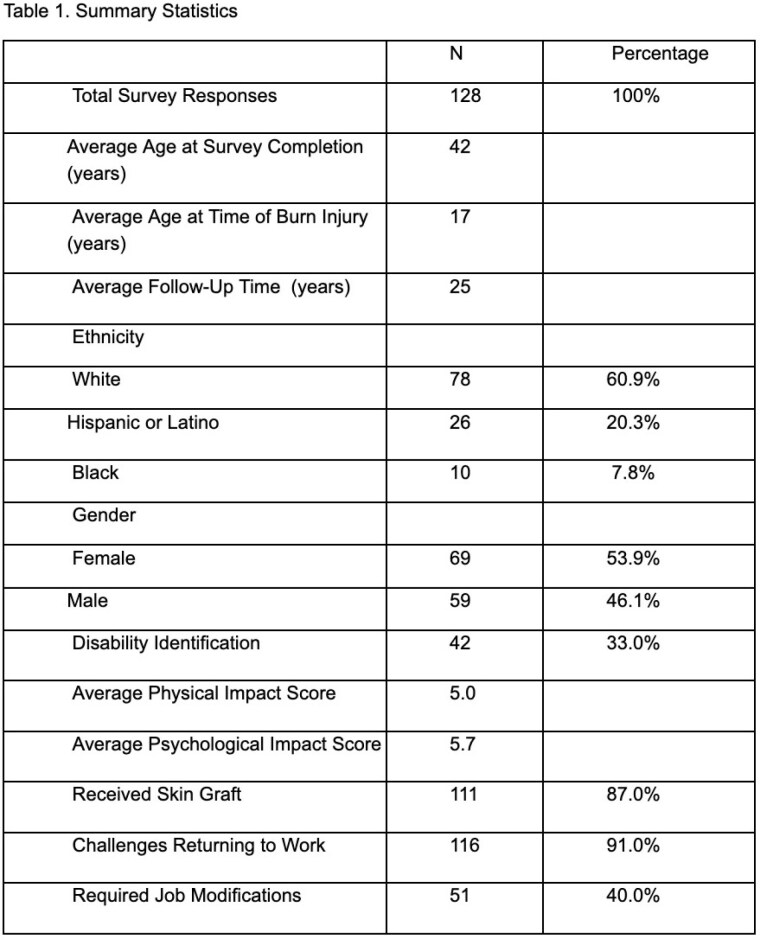# 70 Burn Survivor’s Perspective on Disability: Views and Challenges

**DOI:** 10.1093/jbcr/iraf019.070

**Published:** 2025-04-01

**Authors:** Eloise Stanton, Karel-Bart Celie, Daniel Chacon, Cindy Rutter, Haig Yenikomshian

**Affiliations:** University of Southern California; University of California Keck School of Medicine; Alisa Ann Ruch Burn Foundation; Southern California Burn Model System; University of California Keck School of Medicine

## Abstract

**Introduction:**

Burn injuries can result in profound, long-lasting effects that span physical, psychological, and social domains. Traditionally, research on long-term outcomes for burn survivors has concentrated on measurable physical impairments, psychological trauma, and difficulties in resuming work. However, there is a notable gap in understanding how survivors themselves perceive these impacts, especially concerning their sense of disability and priorities for ongoing research. This study aims to bridge this gap by directly querying burn survivors to gather their perspectives on disability and identify key areas of research that should be prioritized.

**Methods:**

A cross-sectional anonymous survey targeting burn survivors was conducted from January to April of 2023. The survey gathered data on demographics, burn injury characteristics (location, % total body surface area (TBSA), visibility of burn scars), and long-term functional status in the context of their burn injury. Primary outcomes included physical and psychological impact on a scale of 0-10 and work-related changes/challenges.

**Results:**

A total of 128 survey responses were received after distribution. Survey demographic and summary statistics regarding patients’ burns and burn-related disability can be found in Table 1. Multivariate regression analysis identified that greater %TBSA, older age when burned, visible burns, and facial burn scars were significantly associated with increased odds of disability/self-perception of feeling disabled (p< 0.05). When controlling for covariates, multiple logistic regression demonstrated that older age when burned and facial burns were significant predictors of poorer physical but not psychological impact (p< 0.05). Neither gender nor ethnicity was significantly associated with an increased odds of disability.

**Conclusions:**

This study highlights that a substantial number of burn survivors self-identify as disabled, with key factors such as higher TBSA, older age at injury, visible burns, and facial scars significantly contributing to this perception. Additionally, nearly all respondents indicated a need for work-related modifications due to their injuries; however, many did not receive the necessary accommodations. These findings emphasize the critical need for tailored interventions and comprehensive support systems that address both the disability perception and the practical challenges faced by burn survivors, particularly in the workplace.

**Applicability of Research to Practice:**

These findings emphasize the need for healthcare providers to address both physical and psychological aspects of disability in burn survivors, that considers both functional and social challenges, especially for those with large, visible, or facial burns. Increased advocacy for workplace accommodations is essential to support survivors in maintaining employment and improving their quality of life.

**Funding for the Study:**

N/A